# Living donor liver transplantation for a patient with acute liver failure following thyroid storm: a case report

**DOI:** 10.1186/s40792-023-01786-6

**Published:** 2023-12-01

**Authors:** Kantoku Nagakawa, Akihiko Soyama, Takanobu Hara, Hajime Matsushima, Hajime Imamura, Takayuki Tanaka, Michi Morita, Sakaya Kuba, Tomohiko Adachi, Masaaki Hidaka, Hisamitsu Miyaaki, Satoru Akazawa, Ichiro Horie, Motohiro Sekino, Tetsuya Hara, Shinji Okano, Kazuhiko Nakao, Susumu Eguchi

**Affiliations:** 1 Department of Surgery, Nagasaki University Graduate School of Biomedical Sciences, 1-7-1, Sakamoto, Nagasaki, 852-8501 Japan; 2 Department of Gastroenterology and Hepatology, Nagasaki University Graduate School of Biomedical Sciences, Nagasaki, Japan; 3 Division of Endocrinology and Metabolism, Nagasaki University Graduate School of Biomedical Sciences, Nagasaki, Japan; 4 Department of Anesthesiology and Intensive Care Medicine, Nagasaki University Graduate School of Biomedical Sciences, Nagasaki, Japan; 5 Department of Pathology, Nagasaki University Graduate School of Biomedical Sciences, Nagasaki, Japan

**Keywords:** Graves’ disease, Basedow’s disease, Deceased donor liver transplantation, Multi-organ failure

## Abstract

**Background:**

Thyroid storm can be complicated by liver dysfunction, which may occasionally progress to acute liver failure. We herein report a case of acute liver failure following thyroid storm that was treated with living donor liver transplantation after resuscitation from cardiopulmonary arrest.

**Case report:**

The patient was a woman in her 40 s who had been diagnosed with an abnormal thyroid function. She suffered from fatigue and vomiting, and was found to have consciousness disorder, a fever, and tachycardia with a neck mass. She was diagnosed with thyroid storm and was referred to our hospital. After arrival, she went into cardiopulmonary arrest and veno-arterial extracorporeal membrane oxygenation was initiated. In addition to treatment for thyroid storm with antithyroid drugs, steroids, and plasma exchange, extracorporeal life support was required for 5 days. However, despite improvements in her thyroid function, her liver function deteriorated. We planned living donor liver transplantation for acute liver failure after ensuring the recovery and control of the thyroid function following total thyroidectomy. The donor was her husband who donated the right lobe of his liver. Although she experienced acute cellular rejection after surgery, and other complications—including intra-abdominal hemorrhaging and ischemic changes in the intestine—her liver function and general condition gradually improved. One year after living donor liver transplantation, the patient was in a good condition with a normal liver function.

**Conclusions:**

To our knowledge, this is the first report of living donor liver transplantation in a patient with acute liver failure following thyroid storm. Liver transplantation should be recognized as an effective treatment for acute liver failure following thyroid storm.

## Background

Thyroid storm secondary to thyrotoxicosis is a rare a disease. Thyrotoxicosis is an excess of thyroid hormones that can cause a variety of symptoms, while thyroid storm is a more severe and life-threatening condition that can be complicated with organ failure. The mortality rate is reported to be 10.7% in Japan [1] Thyroid storm is often followed by liver dysfunction [2, 3]. Some cases progress to life-threatening acute liver failure [4]. Thyroid storm complicated with hyperbilirubinemia is associated with increased mortality [1]. Treatment is performed to control thyroid hormone levels and support the liver and other organs.

The 2017 Guidelines for the Management of Thyroid Storm by the Japan Thyroid Association and the Japan Endocrine Society recommend aggressive treatments, including therapeutic plasma exchange for acute liver failure following thyroid storm; however, liver transplantation has not been mentioned [1]. Although liver transplantation is the only effective treatment for deteriorating patients [5], this indication has not been established because of the rarity of the condition. To our knowledge, while there have been cases of acute liver failure complicated by thyrotoxicosis and treated with liver transplantation [6, 7] or thyroid storm after liver transplantation [8], only two cases of liver failure following thyroid storm treated with liver transplantation have been previously reported [9, 10]. Both patients underwent deceased-donor liver transplantation.

We herein report the case of a patient with acute liver failure following thyroid storm that was successfully treated with living donor liver transplantation (LDLT).

## Case presentation

The patient was a woman in her 40 s in whom an abnormal thyroid function had been noted 2 years previously, before her referral to our hospital. She consulted a hospital complaining of fatigue and vomiting. At consultation, she also had consciousness disorder, tachycardia, thyromegaly, increased thyroid blood flow, and hyperthyroidism and was diagnosed with thyroid storm. She was referred to our hospital with administration of a β1-blocker and hydrocortisone.

On arrival in the emergency department, a physical examination revealed the following: blood pressure, 110/80 mmHg; pulse, 220 beats per minute with atrial fibrillation; respiration, 60 breaths per minute; body temperature, 39.6 °C; and Glasgow Coma Scale (GCS), 9. Her blood pressure gradually declined, and she experienced cardiopulmonary arrest. After return of spontaneous circulation, an ultrasound cardiogram showed that the ejection fraction was 47%, the transtricuspid pressure gradient was 32 mmHg, and the diameter of the inferior vena cava during expiration and inspiration was 27 and 22 mm, respectively. Hemodynamics were not sustained, so venoarterial extracorporeal membrane oxygenation (ECMO) was initiated. The laboratory findings were as follows: white cell count, 6400/μL; hemoglobin, 10.7 g/dL; platelet count, 79,000/μL; total bilirubin, 8.4 mg/dL; direct bilirubin, 6.4 mg/dL; aspartate aminotransferase, 145 U/L; alanine aminotransferase, 45 U/L; alkaline phosphatase, 596 U/L; albumin, 2.9 mg/dL; prothrombin time (PT) (%), 30%; free triiodothyronine, 30.1 pg/mL; free thyroxine > 7.77 ng/dL; thyroid-stimulating hormone, < 0.005 μIU/mL; and anti-thyroid-stimulating hormone receptor antibodies, 29.8 IU/mL. Antithyroglobulin and antithyroid peroxidase antibodies were absent. The creatine kinase levels were not elevated. An arterial blood gas analysis on arrival revealed the following: pH, 7.275; pO_2_, 317 mmHg (fraction of inspiratory oxygen of 1.0); pCO_2_, 29.5 mmHg; HCO^3−^, 13.3 mmol/L; base excess, − 12.1 mmol/L; and lactate, 7.4 mmol/L. Serological tests for hepatitis A/B/C, antinuclear antibodies, anti-mitochondrial antibodies, anti-gliadin antibodies, and HIV were negative. The diagnosis was thyroid storm due to Graves’ disease and multi-organ dysfunction, acute liver failure, acute heart failure, acute respiratory failure, acute renal failure, disseminated intravascular coagulation, and disturbance of consciousness. She was admitted to the intensive-care unit (ICU).

During the acute course, the patient received an anti-thyroid drug and underwent therapeutic plasma exchange (TPE) under veno-arterial ECMO. The ECMO duration was 5 days. Although her thyroid function improved, her serum total bilirubin level continued to increase to 38 mg/dl until 8 days after her admission. At this timing, the liver transplant team intervened. Plasma exchange, which was performed twice, improved her bilirubin level, but after TPE it increased continuously to 30 mg/dl again. The patient underwent total thyroidectomy to manage her thyroid function. The operative time was 3 h 25 min. Blood loss was 1290 mL due to inflammation and coagulopathy. Intraoperative transfusion required 4 units of red blood cells, 8 units of fresh frozen plasma, and 10 units of platelets.

On postoperative day 3, cervical swelling appeared and hemostasis was needed. Thyroidectomy reduced her thyroid hormone levels, but her bilirubin level increased to 30 mg/dl and her PT was < 40%. While the patient was under intensive care for multi-organ failure caused by thyroid storm, and her circulation and respiration were gradually improving, liver failure was observed to be progressing. Although liver failure was considered a possible prognostic factor, liver transplantation was not considered feasible in the presence of multiple organ failure and DIC, even though the patient was improving. As a life-saving measure, it was considered important to perform liver transplantation without missing the timing when the conditions of multiple organ failure and DIC improved. Although brain-dead-donor liver transplantation was considered, it was difficult to register the patient when multiple organ dysfunction was present as well. We, therefore, planned to perform LDLT as soon as the patient's condition improved while conducting an evaluation of her husband, who was willing to become a donor, simultaneously with intensive care.

**Fig. 1:**
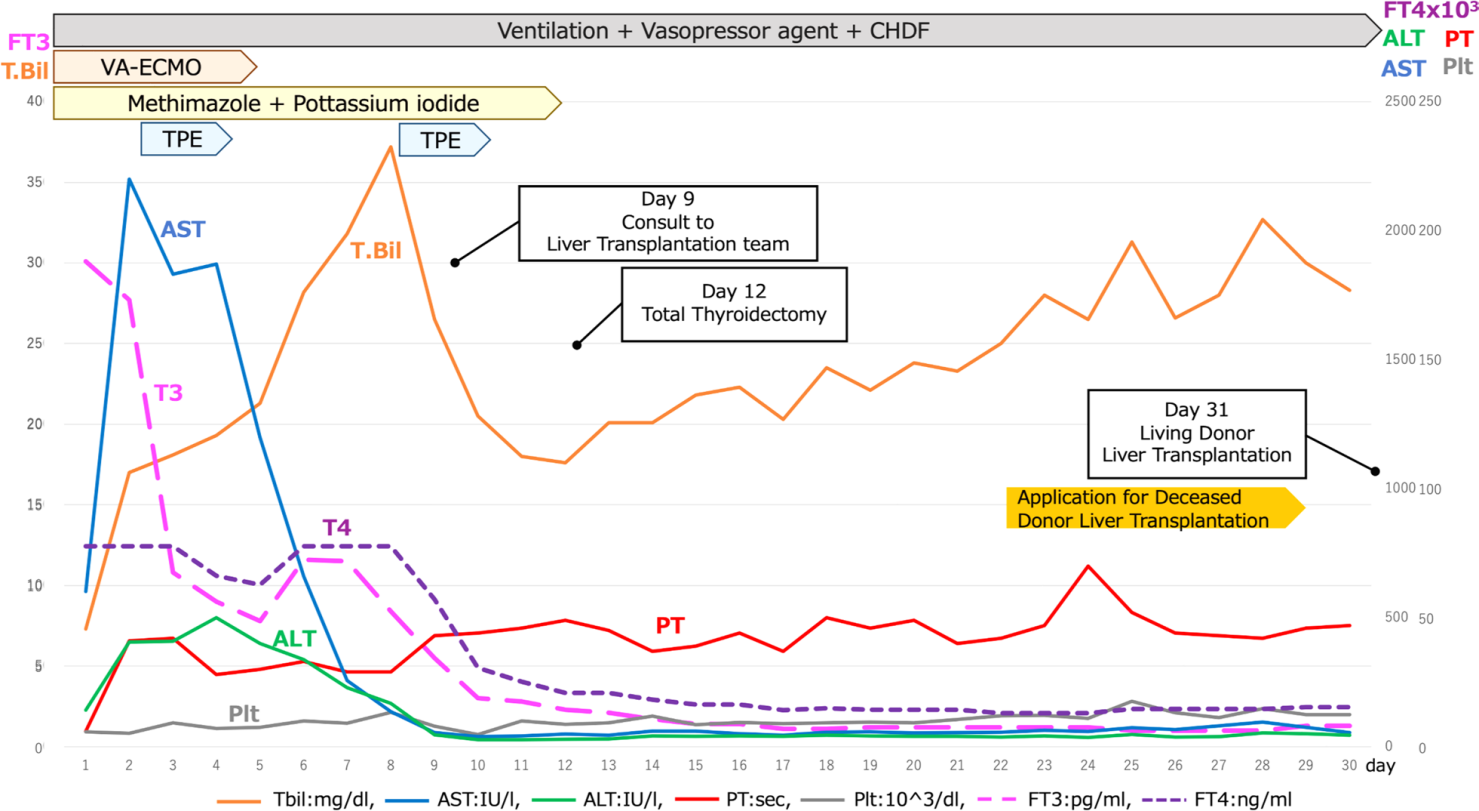
Clinical course from admission to living donor liver transplantation. CHDF: continuous hemodiafiltration; VA-ECMO: veno-arterial extracorporeal membrane oxygenation; TPE: therapeutic plasma exchange; T.Bil: total bilirubin; AST: aspartate transaminase; ALT: alanine aminotransferase; PT: prothrombin time; Plt: platelet; FT3: free triiodo thyronine; FT4: free thyroxine

Her respiration and circulation gradually stabilized under mechanical ventilation, sedation, and a low vasopressor dose. The symptoms were disturbance of consciousness, renal failure, and liver failure. Her consciousness level gradually improved to GCS 10 after tracheostomy. There were no findings of post-resuscitation encephalopathy on magnetic resonance imaging or electroencephalography. Based on these objective findings, the neurologists concluded that the patient’s consciousness was reversible. Her renal failure was controlled stably under continuous hemodiafiltration, and it was possible to switch to intermittent hemodialysis with the expectation of discontinuing dialysis. However, the patient’s liver failure deteriorated. Her Child–Pugh score was 11 with a classification of C, and her model for end-stage liver disease score was 38. Registration as a recipient for deceased-donor liver transplantation was not recommended by the committee because of the expected poor outcome after liver transplantation owing to her severe general condition with multiple organ dysfunction. Since we considered the recovery of her liver function to be mandatory for improving her general status, we decided to perform LDLT as a life-saving treatment. The clinical course of the patient is shown in Fig. 1.

The patient underwent LDLT with a partial liver graft from her husband, whose blood type was A (identical to her blood type) on day 31 of admission. Based on preoperative volumetry, a right lobe graft was transplanted. The weight of the graft was 766 g. The graft weight/recipient standard liver volume ratio was 60.6%. The cold ischemic time was 56 min, and the warm ischemic time was 4 min, with 43 min for anastomosis. Intraoperatively, ischemic change, thought to be caused by shock, catecholamine use, and use of ECMO and continuous hemodiafiltration, was found at the terminal ileum to the ascending colon after the opening of abdomen and worsened during the surgery. Right hemicolectomy was performed after reconstruction of the hepatic artery. Considering the influence of further invasiveness on her general condition, we finished the operation without biliary reconstruction under open abdominal management. Biliary reconstruction was planned to be performed after the patient’s general condition had been stabilized by intensive care. The total operative time was 767 min, and blood loss during the whole operation was 3875 g.

Bile duct reconstruction was performed on post-operative day (POD) 2 as a secondary procedure. The extracted liver weighed 800 g and exhibited a diffuse brown–green color change. A histological examination revealed acute chronic changes. Bile thrombi were found in the centrilobular bile canaliculi, and hepatocytes and Kupffer cells showed pigmentation with a high degree of hepatocyte necrosis and neutrophil infiltration, indicating significant acute changes. In addition, there were chronic findings, such as enlargement of the bile canaliculi, fibrosis in the portal and periportal regions, enlargement of hepatocytes mainly in the peripheral region, and Mallory–Denk body formation, suggesting that the liver with thyroid hormone-induced chronic hepatitis and chronic bile congestion had changed acutely due to thyroid crisis. No evidence of liver cirrhosis was found.

**Fig. 2:**
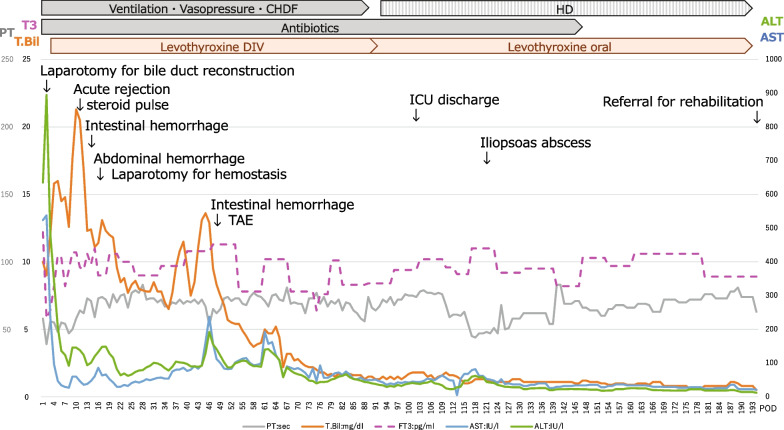
Clinical course from living donor liver transplantation to discharge. CHDF: continuous hemodiafiltration; ICU: intensive care unit,

Regarding immune suppression therapy, methylprednisolone and basiliximab were administered, given her renal failure. However, the patient’s condition was complicated by acute cellular rejection, intra-abdominal hemorrhaging, and colonic hemorrhaging. A sharp increase in liver enzymes was observed on POD 9, so a liver biopsy was performed with suspicion of rejection. A histological examination revealed a diagnosis of acute cellular rejection, and the patient was treated with steroid pulse therapy and a continuous infusion of the tacrolimus, resulting in improvement. The trough value of tacrolimus was controlled at around 15 ng/ml. At the time of the liver biopsy, the intercostal artery was injured, which was complicated by intra-abdominal bleeding on POD 15; therefore, emergency laparotomy for hemostasis was performed. Gastrointestinal hemorrhaging was observed on POD 23, and ischemic colitis was diagnosed via endoscopy. After conservative treatment, the ischemic colitis improved, but bleeding occurred again on POD 53, and transcatheter arterial embolization of the inferior mesenteric artery was performed to achieve hemostasis. After these complications, the patient’s liver function and general condition gradually improved. The clinical course after liver transplantation is shown in Fig. 2.

Two years after LDLT, other than the need for intermittent hemodialysis, the patient is in good condition with a normal liver function and lives her daily life. In addition, the donor’s postoperative course was uneventful. The patient was discharged on POD 10.

## Discussion

The mechanism of liver damage caused by thyroid storm has both direct and indirect effects. Relative oxygen deficiency due to increased systemic oxygen consumption, inhibition of lactate metabolism in the liver by thyroid hormones, congested liver due to heart failure, and decreased hepatic blood flow due to hypotension all lead to oxygen deficiency and ischemia of the liver. Other factors include hepatotoxicity caused by antithyroid drugs, induction of autoimmune hepatitis, and exacerbation of underlying liver diseases [10, 11]. Antithyroid drug-induced hepatotoxicity occurs in less than 0.5% of patients [12] and is more common with propylthiouracil than with methimazole. [13] Methimazole-induced hepatotoxicity is typically cholestatic, while propylthiouracil-induced hepatotoxicity is typically hepatocellular. [14, 15] Liver damage caused by an abnormal thyroid function has been biochemically proven, and caspases, known to be markers of apoptosis, are increased in hyperthyroid rats and decreased in hypothyroid rats, proving that hepatocyte apoptosis progresses in hyperthyroid rats [16]. Pathologically, it was also reported that lobular central necrosis and sinusoidal congestion were observed as a result of circulatory disorder, and centriolobular to sub-massive necrosis was observed as a result of hepatocellular damage [10].

Regarding the frequency, increasing aspartate aminotransferase and alanine aminotransferase levels are reported in 27% and 37% of hyperthyroid patients, respectively [17]. Regarding liver damage due to thyroid storm in particular, 68% of patients with thyroid storm seem to have gastrointestinal symptoms, while jaundice has been reported in 20% of all patients [18]. When the total bilirubin level is > 3.0 mg/dl, the mortality rate of thyroid storm associated with hyperbilirubinemia is reported to be 32.3%, and the bilirubin value is reported to be an important indicator of liver necrosis and a prognostic factor [18, 19]. Based on these reports, guidelines for the treatment of thyroid storm recommend aggressive treatment for acute liver failure due to thyroid storm, although there is no statement about liver transplantation.

To date, only two cases of liver transplantation for liver failure following thyroid storm have been reported [9, 10]. Both were associated with hyperthyroidism in young women who underwent deceased donor liver transplantation with a high bilirubin level immediately before transplantation. In both cases, total thyroidectomy was performed prior to transplantation. There was no description of other organ failure, but normalization of the thyroid hormone levels was mentioned in the first case (Table 1).

**Table 1:** Characteristics of reported cases

	Case	Pre-existing thyroid disease	Other organ failure	Treatment for thyroid storm	Thyroid hormone just before LT	Outcome
Hambleton et al. [6]	22 y.o Female	Pregnancy-related Graves’ disease	None	Thyroidectomy	Normalized	Alive
de Campos Mazo et al. [7]	19 y.o Female	Hyperthyroidism	None	Plasmapheresis Thyroidectomy	N/A	Alive
Our case	40s Female	Basedow’s disease	Heart failure Renal failure Post-cardiac arrest	Plasmapheresis Thyroidectomy	Improved	Alive

T-Bil, total bilirubin; LT, liver transplantation; N/A, not available

In the present case, LDLT was performed, and the patient was rescued by liver transplantation even after cardiopulmonary arrest. In Japan, LDLT is generally performed more often than deceased-donor liver transplantation due to the low number of organ donations (about 1 donor/per million population), and it is often associated with a long waiting time [20]. Therefore, LDLT should always be considered for patients with acute liver failure. The indications for liver transplantation in patients with liver failure caused by thyroid storm remain unclear, as the number of reported cases is limited. Considering the indication for common acute liver failure, it is important that accepted criteria associated with a poor prognosis be persistent and that co-morbidities independent of acute liver failure that would impact the survival and complications of acute liver failure associated with reduced survival be absent [21].

As mentioned above, when the liver transplantation team was consulted regarding the present patient on day 8 of admission, the bilirubin value was > 38 mg/dl, which is significantly higher than the values that have been shown to be associated with a poor prognosis [18, 19]. In fact, we had been contemplating liver transplantation from the time of intervention, but it was challenging to ascertain whether or not the patient’s multiorgan failure after cardiac arrest, especially consciousness disorder, was irreversible. Prior to performing liver transplantation, our team needed to confirm that the patient’s cardiac failure had improved, that there was no irreversible cerebral damage, and that the disseminated intravascular coagulation had been resolved. It took some time to establish these clinical findings.

**Fig. 3:**
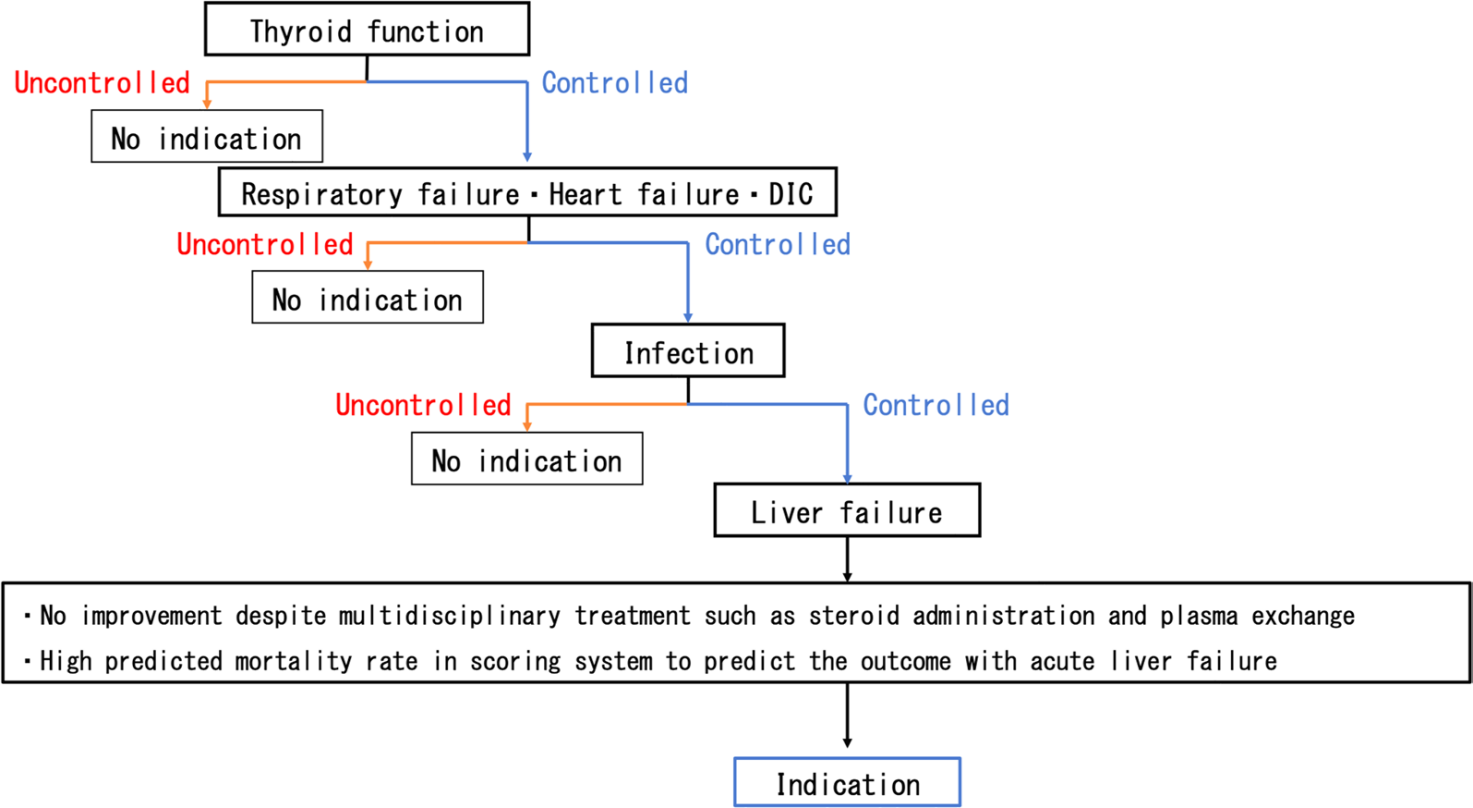
Indications for liver transplantation for acute liver failure following thyroid storm. DIC: dissemination intravascular coagulation

On the premise that thyroid function is controlled without extremely severe respiratory distress, cardiac failure, or uncontrolled infection, it seems reasonable to consider liver transplantation for patients with continuously deteriorating liver dysfunction and a high predicted mortality rate whose liver function does not improve despite medical multidisciplinary treatment (e.g., steroid administration and plasma exchange) (Fig. 3). Regarding the rejection, it was reported that TSH levels were found to be increased with acute rejection [22]. However, in the present case, the TSH levels were low at the timing of acute rejection. The relationship between the thyroid function and acute rejection was thus unclear in our case.

## Conclusions

Based on our experience, in patients with liver failure following thyroid storm whose condition remains severe even after cardiopulmonary resuscitation, liver transplantation at the appropriate time may be an effective life-saving treatment option that should be considered early in the course of treatment.

## Data Availability

All data generated or analysed during this study are included in this article. Further enquiries can be directed to the corresponding author.

## Abbreviations

GCS: Glasgow coma scale
ECMO: Extracorporeal membrane oxygenation
PT: Prothrombin time
TPE: Therapeutic plasma exchange
LDLT: Living donor liver transplantation
POD: Post-operative day
